# Total phenolic, flavonoid, alkaloid and iridoid content and preventive effect of Lider-7-tang on lipopolysaccharide-induced acute lung injury in rats

**DOI:** 10.1590/1414-431X20175916

**Published:** 2017-10-19

**Authors:** Ch. Erdenechimeg, A. Guiqide, B. Dejidmaa, Ch. Chimedragchaa, S. Purevsuren

**Affiliations:** 1 Institute of Traditional Medicine and Technology, Ulaanbaatar, Mongolia; 2 The Inner Mongolia Autonomous Region International Mongolian Hospital, HuhHot, Inner Mongolia, China; 3 School of Pharmacy, Mongolian National University of Medical Sciences, Ulaanbaatar, Mongolia

**Keywords:** Traditional medicine, Lider-7-tang, Phenolic, Acute lung injury, Lipopolysaccharide

## Abstract

Lider-7-tang, a medicine used for the treatment of respiratory diseases especially pneumonia and fever in Mongolian Traditional Medicine, was selected for this phytochemical and pharmacological study. The objectives of the study were to determine total biological active substances and analyze the effects of Lider-7-tang treatment in rats with acute lung injury (ALI). Quantitative determination of the total active constituents (phenolic, flavonoid, iridoid and alkaloid) of the methanol extract of Lider-7-tang was performed using Folin-Ciocalteu reagent, aluminum chloride reagent, Trim-Hill reagent, and Bromocresol green reagent, respectively. A total of fifty 8–10-week-old male Wistar rats (200–240 g) were randomized into three groups: control group, lipopolysaccharide (LPS) group (7.5 mg/kg) and LPS+Lider-7 group (90 mg/kg Lider-7-tang before LPS administration). The total content of alkaloids was 0.2±0.043%, total phenols 7.8±0.67%, flavonoids 3.12±0.206%, and iridoids 0.308±0.0095%. This study also evaluated the effects of Lider-7 on levels of inflammatory mediators by observing histopathological features associated with LPS-induced ALI. The rats pretreated with Lider-7 had significantly lower levels of IL-6 (at 3 and 6 h), and TNF-α (at 3, 6, 9, and 12 h). The current study showed that Lider-7 exerted a preventive effect against LPS-induced ALI, which appeared to be mediated by inhibiting the release of pro-inflammatory cytokines.

## Introduction

Acute lung injury (ALI) is an acute inflammatory disease, characterized by excess production of inflammatory factors in lung tissue, and followed by non-cardiogenic dyspnea, severe hypoxemia, and pulmonary edema, thus leading to both high morbidity and mortality (1,2). A major cause of the development of ALI is sepsis, wherein Gram-negative bacteria are a prominent cause (3). The intraperitoneal injection of lipopolysaccharide (LPS), a component of the outer cell wall of most Gram-negative bacteria, mimics human Gram-negative ALI and is one of the most commonly accepted models for ALI (4). Lipopolysaccharide, binding to its receptor, toll-like receptor 4, provokes the activation of a key pro-inflammatory transcription factor, nuclear factor κB, which induces the expression of various pro-inflammatory cytokines and chemokines, such as tumor necrosis factor-α (TNF-α), interleukin-1β, and macrophage inflammatory protein-2 (5). As a consequence of the strong inflammatory response, alveolar structures change, endothelial and alveolar permeability increase and alveolar fluid clearance decreases, thus critically impairing lung function (3,6).

Lider-7-tang is one of the traditional Mongolian herbal medicines consisting of seven herbs, Radix *Sophoroe alopecuroides*, Radix *Inulae helenium*, Fructus *Gardeniae*, Fructus *Terminaliae billericae*, Fructus *T. chebulae*, Herba *Gentianae barbatae* and Herba *Lagotis integrifoliae*. Lider-7-tang has been used to treat cold and flu symptoms such as nasal congestion, headache, body ache, fever, sore throat pain, and cough for a long time in Traditional Mongolian Medicine (7,8). Lider-7-tang has a light green color, has an odor, and tastes bitter, smooth, fatty and soft.

*S. alopecuroides* L. shows a wide spectrum of pharmacological activities, including detoxification, anti-bacterial, anti-inflammatory, pain killing, asthma cough, and anti-tumor, among others (9–12). There are many chemical constituents in *S. alopecuroides* L., and the main bioactive components of this plant are alkaloids, flavones, volatile oils, and quinones. In the 1980's, there were more than 20 kinds of alkaloids isolated and identified from *S. alopecuroides* L., such as sophocarpine, matrine, oxymatrine, sophoridine, sophoramine etc. (13,14). *S. alopecuroides* L. contains quercetin, rutoside, isobavachin, glabol, trifolirhizin, ammthamnidin, vexibinol and vexibidin (14). Three new flavonostilbenes (alopecurones M–O) were isolated from the root bark of *S. alopecuroides* L. together with 21 known compounds. All isolates were evaluated for their potential to inhibit LPS-induced nitric oxide production in RAW 264.7 cells (15). *S. alopecuroides* L. has a great effect as an anti-inflammatory. The main effective substances associated with anti-inflammatory activity are considered the alkaloids of *S. alopecuroides* L. (11).

*I. helenium* L. has been investigated for pharmacological benefits including antioxidant and anti-inflammatory activities, hepatoprotective characteristics, cytotoxicity, and antimicrobial properties (16–18). Chemical analysis of the rhizome and roots showed that *I. helenium* contains many bioactive compounds including polysaccharide inulin (up to 44%), essential oil with eudesmane-type sesquiterpene (up to 5%), lactones (mainly alantolactone and isoalantolactone), thymol derivatives, terpenes, and sterols (19,20).

Flavonoids are a group of polyphenolic compounds and exhibit several biological effects such as anti-hepatotoxic, anti-inflammatory and anti-ulcer activity. All ingredients of Lider-7-tang contain flavonoids and phenolic compounds. For example, 5,7,3',4'-tetrahydroxyflavone, doismetin, apigenin, chrysoeriol, tilianin and luteolin, etc. have been isolated from *G. barbatae* L (14). There are iridoid glycosides *Gardenia jasminoides* Ells and *L. integrifolia*. *G. jasminoides* extracts and their main active phytoconstituents geniposide, genipin, crocin, crocetin have been reported for a wide range of pharmacological activities such as anti-hyperglycemic, anti-atherosclerotic, anti-inflammatory, anti-arthritis, and anti-cancer etc. (21,22).

Gallic acid is a polyphenolic compound with antioxidant property. Gallic acid, a major constituent of *T. bellirica* (Barur), *T. chebula* (Arur), is useful for common colds and fever and has diuretic, laxative, liver tonic, refrigerant, stomachic, restorative, alterative, antipyretic, and anti-inflammatory effects (23,24).

Therefore, we postulated that Lider-7-tang could protect against LPS-induced lung injury. In the present study, we tested this hypothesis using a rat model of LPS-induced ALI.

## Material and Methods

### Plant materials

The crude herbal medicines from *S. alopecuroides, I. helenium, T. chebula, T. bellerica, G. jasminoides* were purchased from Traditional Drug Factory at the Institute of Traditional Medicine and Technology (Mongolia). *G. barbatae* and *L. integrifolia* were collected from Khuvsgul, Mongolia in 2015. The origin of each herbal medicine was taxonomically confirmed by Prof. Ganbold E (Ulaanbaatar University, Ulaanbaatar, Mongolia).

### Ethics statement

All experimental procedures performed in this study were in accordance with the Guide for the Care and Use of Laboratory Animals, proposed by the Institute of Traditional Medicine and Technology. The study protocol was approved by the Biomedical Ethics Subcommittee of Mongolian National University of Medical Sciences, Mongolia.

### Experimental animals

A total of fifty 8–10-week-old male Wistar rats (200–240 g) were used in this study. All experimental animals were obtained from the Experiment Animal House, Institute of Traditional Medicine and Technology. The rats were housed in cages and maintained at room temperature with a 12-h light/dark cycle. They were fed with standard pellet diet and tap water *ad libitum*.

### Reagent

Standards of gallic acid, rutin, oxymatrine and aucubin were obtained from Sigma-Aldrich (USA). Folin Ciocalteu’s phenol reagent and aluminum chloride (AlCl_3_) of Sangon (China) were used in the study. All other solvents and chemicals were of analytical grade.

*Escherichia coli* 055:B5 endotoxin from Sigma-Aldrich and the cytokine immunoassay kits from Shanghai MLBIO Biotechnology Co. Ltd. (China) were used in the study.

### Chemical analysis

#### Sample preparation

Powdered medicine was precisely weighed (1.0 g), extracted with 50 mL of 70% ethanol in reflux for 30 min, and filtrated. The supernatant was used as the test solution.

#### Estimation of total flavonoid contents

The solution was treated with 1 mL of 5% NaNO_2_, 1 mL of 10% Al(NO_3_)_3_ and 10 mL of 4% NaOH solution, and absorbance values were determined using a spectrophotometer (UNICO UV-2102 C, China) at 500 nm. The content of flavonoids in extracts is reported as rutin equivalent (mg of RU/g of extract) (25).

#### Estimation of total polyphenolic compounds

The amount of total phenolics was determined using the Folin-Ciocalteu assay. The Folin-Ciocalteu reagent (diluted 1:10 in water) and aqueous Na_2_CO_3_ (10.75%) were successively added to the extract. In 30 min, the absorbance value was measured at 760 nm. Gallic acid was used to establish the calibration curve, and total polyphenolic content is reported as g/kg (26).

#### Determination of total alkaloids

Total alkaloids were determined by the spectrophotometric method based on the reaction with bromocresol green (69.8 µg/mL) and absorbance was measured at 420 nm. Oxymatrine was used to establish the calibration curve, and total alkaloids content is reported as oxymatrine equivalent as g/kg (27).

#### Determination of total iridoids

The content of iridoids was determined according to the colorimetric method based on a Trim-Hill reaction. Each extract (0.4 mL) was mixed with 4 mL of Trim-Hill reagent (acetic acid-0.2% CuSO_4_-conc. HCl, 10:1:0.5), afterward absorbance was measured at 609 nm, and the blue color indicated the presence of iridoids. The amount of iridoids was calculated using aucubin (0.1–1 mg/mL) calibration curve. Results are reported as the mean value of 3 replicates (28).

### Preventive effect of Lider-7-tang on LPS-Induced ALI in rats

#### Experimental protocols

Rats were randomized into three groups: control group (n=10), LPS group (n=20), in which LPS (7.5 mg/kg dissolved in 0.5 mL sterile saline) was administered by an intravenous injection (*iv*) via the tail vein; and LPS+Lider-7 group (n=20), in which Lider-7 (90 mg/kg, orally) was administered 30 min before injection of LPS (7.5 mg/kg dissolved in 0.5 mL sterile saline, *iv*) orally. Rats were euthanized with an overdose of sodium pentobarbital (100 mg/kg, *ip*). Lung tissue specimens and blood samples were then obtained for further analysis (29).

### Histological analysis

Twelve hours after LPS administration, the rats were euthanized (n=5, 3, and 5 in the control, LPS, and LPS+Lider-7 groups, respectively). The obtained lung tissue specimens were fixed with 10% formalin, embedded in paraffin, cut into 5-mm thick sections and mounted onto slides. The sections were then stained with hematoxylin and eosin (H&E) according to the standard staining method (30). Histologic changes were graded by a pathologist blind to the clinical status of the rats. Then the lung tissue samples were scored for the degree of intra-alveolar edema, intra-alveolar hemorrhage, and neutrophil infiltration using grades 0 to 4 (0, absent; 1, mild; 2, moderate; 3, severe; 4, overwhelming) with a maximum score of 12, as described previously (31).

### Wet-to-dry weight ratio

After the animals were euthanized at 12 h, the chest cavity was opened and the right lung was ligated and excised. The lung specimen was then briefly rinsed in phosphate buffered saline (PBS), blotted, and weighed to determine the ‘wet’ weight. Subsequently, the lungs were dried in an oven at 80°C for 24 h to obtain the dry/weight. The ratio of wet-to-dry (W/D) weight was then calculated.

### Plasma levels of cytokines (TNF-α and IL-6)

Blood samples were collected via cardiac puncture at 3, 6, 9, and 12 h after the administration of LPS and from healthy rats. All rats were euthanized with phenobarbital sodium before blood collection. The collected blood samples were centrifuged at 377.3 *g* for 10 min at 4°C, and the plasma supernatant was stored at –20°C until further analysis. The plasma levels of TNF-α and IL-6 were detected using solid-phase sandwich enzyme-linked immune sorbent assay (ELISA, Shanghai MLBIO Biotechnology Co. Ltd.) kits specific for the detection of these factors, and the absorbance was measured at 450 nm by a plate reader (Chromate 4300 microplate, Shanghai MLBIO Biotechnology Co. Ltd., China).

### Statistical analysis

Data are reported as means±SD. Statistical significance was determined by one-way analysis of variance followed by Tukey’s multiple comparison test. A P value <0.05 was considered statistically significant.

## Results

### Total phenolic, flavonoid, alkaloid and iridoid contents

The flavonoid contents of the extract in terms of rutin equivalent (standard curve equation: y = 11.815x – 0.0092, r^2^ = 1000) were from 4.0 to 40.0 (Table 1). The flavonoid content in the extract of Lider-7-tang was 31.2±2.06 mg/g. Table 1 also shows the content of total phenols reported as gallic acid equivalent (standard curve equation: y =110.77 x – 0.0736, r^2^ = 0.995), which were from 0.72 to 2.1 µg/mL. Total phenol was 78.0±6.7 mg/g in the Lider-7-tang. The content of iridoids in term of aucubin equivalent (standard curve equation: y = 9.5981 x + 0.0132, r^2^ = 0.966) were between 3–18 µg/mL. Iridoid content was 3.08±0.095 mg/g in Lider-7-tang extract. The content of alkaloids was measured in term of oxymatrine equivalent (the stander curve equation: y = 5.5435 x + 0.0613, r^2^ = 0.957) and determined to be from 4.0 to 50.0 µg oxymatrine per mL of chloroform. The total alkaloids were determined to be 1.6±0.43 mg/g in Lider-7-tang extract (Table 1).

**Table 1.** Total phenolics, flavonoids, alkaloids and iridoids in methanol extracts of the Lider-7-tang (n=3).

Bioactive substance	mg/g dry mass
Flavonoids	31.2±2.06
Total phenolics	78.0±6.7
Total alkaloids	1.6±0.43
Iridoids	3.08±0.095

### Lung preventive effect of Lider-7

#### Lider -7 pre-treatment decreased LPS-induced pathological changes in lung tissue

**Figure 1.**
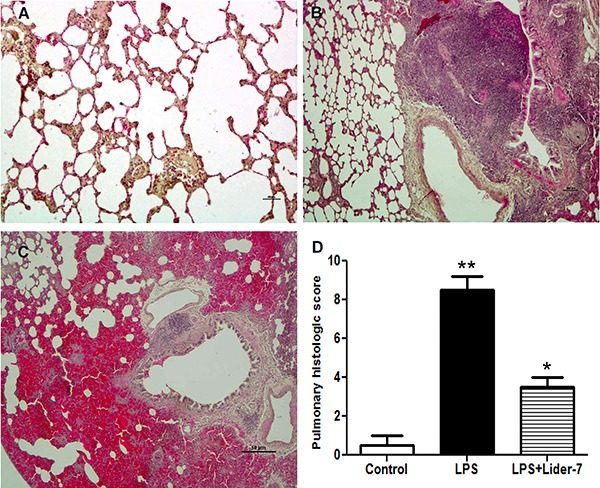
Histopathological changes in lung tissue samples of the three groups. Hematoxylin and eosin (×200 magnification). *A*, Control group with normal lung structure. *B*, Lipopolysaccharide (LPS) group with increased alveolar wall thickness, edema, bleeding and infiltration of inflammatory cells. *C*, LPS+Lider-7 group showed less structure destruction and inflammatory infiltration. *D*, Comparison of the pulmonary histological scores of the three groups. Data are reported as means±SD. *P<0.05, LPS+Lider-7 group compared to control group; **P<0.001, LPS group compared to control group (one-way ANOVA).

The control group showed no significant histological alterations. The LPS group showed increased alveolar wall thickness, edema, bleeding and infiltration of inflammatory cells at 12 h after LPS administration, indicating the occurrence of bronchopneumonia or ALI. Rats pre-treated with Lider-7 showed significantly less inflammation and change of pulmonary structure, normal alveolar majority air space and hyperplasia of lymphoid cells after LPS administration compared to those not treated with Lider-7 (Figure 1A–C). The total scores of the histological changes in the groups indicated that the degree of pulmonary injury or bronchopneumonia in the LPS+Lider-7 group was significantly less than in the LPS group (P<0.05, Figure 1D)

#### Effect of Lider-7 pre-treatment on right lung W/D ratio

**Figure 2.**
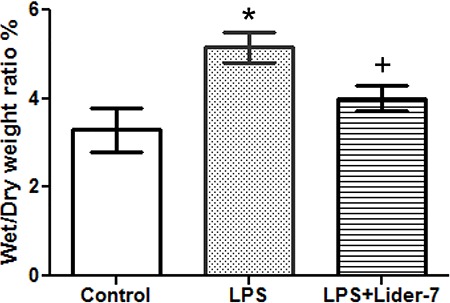
Comparison of the wet/dry ratio. The extent of pulmonary edema was assessed using the wet/dry ratio at 12 h after lipopolysaccharide (LPS) infusion. Control group: n=5; LPS group: n=3; LPS+Lider-7 group: n=5. Data are reported as means±SD. *P<0.05, LPS group compared to control group; ^+^P<0.05, LPS+Lider-7 group compared to LPS group (ANOVA).

The LPS group had a significantly higher W/D ratio than the healthy group, indicating the presence of pulmonary edema (P<0.05). However, the W/D ratio in the LPS+Lider-7 group was significantly decreased compared to the LPS group, indicating that Lider-7 attenuated the degree of pulmonary edema induced by LPS (P<0.01; Figure 2).

#### Effect of Lider-7 on the expression of pro-inflammatory cytokines of plasma

**Figure 3.**
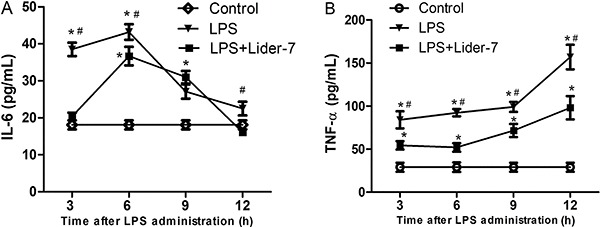
Changes in the levels of pro-inflammatory cytokines. *A,* Interleukin (IL-6); *B,* tumor necrosis factor (TNF α). Control group: n=5 for each time point; lipopolysaccharide (LPS) group: n=5 (3 and 6 h), n=4 (9 h) and n=3 (12 h); LPS+Lider-7 group: n=5 for each time point. Data are reported as means±SD. *P<0.05, LPS and LPS+Lider-7 groups compared to control group; ^#^P<0.05, LPS group compared to LPS+Lider-7 group (ANOVA).

In the LPS group, the levels IL-6 significantly increased after LPS administration and reached peak levels at 6 h. Thereafter, the levels decreased gradually to baseline at 12 h. However, the levels of the late stage pro-inflammatory cytokine TNF-α increased gradually and reached a peak at 12. In contrast, the rats pretreated with Lider-7 had significantly lower levels of IL-6 (LPS+Lider-7 group *vs* LPS group: P<0.05 at 3, 6 and 12 h) and of TNF-α (P<0.05 at 3, 6, 9, and 12 h) (Figure 3).

## Discussion

The median lethal dose for Lider-7 tang was determined as 8.9 g/kg on the result of acute toxicity studies carried out by the express method of Prozorovskii et al. (32). Therefore, we selected the dose of 90 mg/kg for this study. In the present study, a rat model of ALI was successfully established by the intravenous administration of LPS. We found that LPS exposure caused a dramatic increase in the W/D ratio, reflecting the pulmonary edema. Furthermore, histopathological analysis revealed a loss of epithelial integrity. Taken together, these manifestations confirmed the development of LPS-induced ALI. Interestingly, pretreatment with Lider-7 reduced the extent of histopathological changes and secretion of pro-inflammatory cytokines in rat lung tissue.

Gram-negative sepsis is the most common risk factor of acute respiratory distress syndrome. LPS is the principal component of the outer membrane of gram-negative bacteria and is a potent stimulator of rapid pro-inflammatory cytokine production. The elevated expression of TNF-α and IL-6 is an important step in the pathogenesis of ALI and acute respiratory distress syndrome (33). Many natural substances such as sophoraflavanone G (34), quinolizidine alkaloids (35–38), alantolactone (16–20) and geniposide (21) have shown the effect of decreasing LPS-induced inflammation via suppression of pro-inflammatory cytokine secretion.

Because *S. alopecuroides* is the main compound in Lider-7-tang, we speculate that quinolizidine alkaloids had a major contribution to the effects observed. Moreover, sesquiterpene lactones have shown anti-inflammatory effects, so they might have assisted in the effects observed.

Consistently, our study showed that the levels of TNF-α and IL-6 reached a peak at 6 h after LPS administration and then returned to baseline levels. The persistence of lung injury suggests that other late stage downstream pro-inflammatory cytokines may be involved in the progression of ALI.

The current study demonstrated that Lider-7-tang 1) ameliorated histopathological changes that indicate lung injury, and 2) inhibited the release of pro-inflammatory cytokines in rats with ALI. Taken together, these results suggest that Lider-7-tang might be a potential candidate for the pre-treatment of LPS-induced ALI.
